# Functional characterization of an ornithine cyclodeaminase-like protein of *Arabidopsis thaliana*

**DOI:** 10.1186/1471-2229-13-182

**Published:** 2013-11-18

**Authors:** Sandeep Sharma, Suhas Shinde, Paul E Verslues

**Affiliations:** 1 Institute of Plant and Microbial Biology, Academia Sinica, No. 128 Sec. 2 Academia Rd, Nankang Dist., Taipei 11529, Taiwan

**Keywords:** Ornithine cyclodeaminase, Proline, Drought, *Arabidopsis thaliana*

## Abstract

**Background:**

In plants, proline synthesis occurs by two enzymatic steps starting from glutamate as a precursor. Some bacteria, including bacteria such as *Agrobacterium rhizogenes* have an Ornithine Cyclodeaminase (OCD) which can synthesize proline in a single step by deamination of ornithine. In *A. rhizogenes, OCD* is one of the genes transferred to the plant genome during the transformation process and plants expressing *A. rhizogenes* OCD have developmental phenotypes. One nuclear encoded gene of *Arabidopsis thalian*a has recently been annotated as an *OCD* (*OCD-*like*;* referred to here as *AtOCD*) but nothing is known of its function. As proline metabolism contributes to tolerance of low water potential during drought, it is of interest to determine if *AtOCD* affects proline accumulation or low water potential tolerance.

**Results:**

Expression of *AtOCD* was induced by low water potential stress and by exogenous proline, but not by the putative substrate ornithine. The AtOCD protein was plastid localized. T-DNA mutants of *atocd* and *AtOCD* RNAi plants had approximately 15% higher proline accumulation at low water potential while *p5cs1-4/atocd* double mutants had 40% higher proline than *p5cs1* at low water potential but no change in proline metabolism gene expression which could directly explain the higher proline level. *AtOCD* overexpression did not affect proline accumulation. Enzymatic assays with bacterially expressed AtOCD or AtOCD purified from *AtOCD:Flag* transgenic plants did not detect any activity using ornithine, proline or several other amino acids as substrates. Moreover, *AtOCD* mutant or over-expression lines had normal morphology and no difference in root elongation or flowering time, in contrast to previous report of transgenic plants expressing *A. rhizogenes OCD*. Metabolite analysis found few differences between *AtOCD* mutants and overexpression lines.

**Conclusions:**

The Arabidopsis OCD-like protein (AtOCD) may not catalyze ornithine to proline conversion and this is consistent with observation that three residues critical for activity of bacterial OCDs are not conserved in AtOCD. *AtOCD* was, however, stress and proline induced and lack of *AtOCD* expression increased proline accumulation by an unknown mechanism which did not require expression of *P5CS1*, the main enzyme responsible for stress-induced proline synthesis from glutamate. The results suggest that AtOCD may have function distinct from bacterial OCDs.

## Background

Proline (Pro) metabolism has a number of unique functions in plants as well as microorganisms [1,2]. Bacteria use proline as a compatible solute to osmoregulate and prevent injury from osmotic stress [3]. Proline accumulation functions in plant osmotic adjustment and influences plant development [1,4-8] as well as buffering of cellular redox status during abiotic stress [1,2,9]. Proline metabolism can also act as an inducer of the hypersensitive response during plant pathogen infection [10,11]. This latter function is likely caused by proline catabolism promoting the formation of reaction oxygen in the mitochondria or by a yet poorly understood role of ∆^1^-pyroline-5-carboxylate (P5C), the main intermediate of both proline synthesis and catabolism. A similar role of proline catabolism and P5C in apoptosis and tumor suppression and additional roles of proline metabolism in tumor metabolism have been proposed in mammals [7,12,13]. These examples illustrate how there are a number of reasons that proline metabolism, and potential new genes related to proline metabolism, are of significant interest.

The core components of plant proline metabolism and their role in plant stress responses have been examined in a number of studies. Proline is mainly derived from glutamate through two enzymatic steps which in *Arabidopsis thaliana* are catalyzed by P5C synthetase1 (P5CS1) and P5CS2 which convert Glu to the intermediate P5C and P5C reductase (P5CR) which converts P5C to proline [1,2]. P5CS1 expression is up-regulated under abiotic stress [1,2,14], and *p5cs1* mutants have greatly reduced proline accumulation. P5CS1 is required for tolerance of low water potential [9] and salt stress [11]. Mutants of P5CS2 have greatly reduced viability indicating that P5CS2 has a greater role in proline synthesis during normal development and an important role in reproductive development [4,7,13]. The subcellular localization of stress induced proline synthesis is still not entirely certain as it is not clear whether P5CS1 is in the chloroplast, associated with the outside of the chloroplast or partially cytosolic and whether its localization changes during stress [11]. P5CR has been found in both cytosol and chloroplast [12,15], although it may be localized predominantly in the cytosol [7]. The conversion of ornithine into P5C by Orn-δ-aminotransferase (OAT) has traditionally been considered as an alternative pathway of proline synthesis. However, OAT has recently been shown to be a mitochondrial enzyme which may not have a major role in proline synthesis [16].

Some bacterial species, including the plant pathogenic bacteria, *Agrobacterium tumefaciens* and *A. rhizogenes*, have another enzyme, ornithine cyclodeaminase (*OCD*), which synthesizes proline in a single step from ornithine [8,17]. The enzymatic reaction requires NAD^+^ as a cofactor and consists of the deamination of L-ornithine into L-proline with ammonium released as a byproduct [17,18]. The enzyme has also been classified as a member of the μ crystalline family based on the sequence similarity to a protein highly expressed in eye lens of marsupials [19]. In *A. tumefaciens,* OCD is localized on the Ti plasmid but not in the transferred DNA (T-DNA) region. It is involved in the last step of the catabolism of ornithine derived (via arginine) from plant-synthesized opines that provide nitrogen and carbon for bacterial growth [18]. In contrast, the OCD of *A. rhizogenes,* is localized in the T-DNA region of the Ri plasmid and is referred to as *RolD*[8]. Deletion of *A. rhizogenes* RolD increased callus formation but roots from the calli grew more slowly than those from calli initiated by the wild type *A. rhizogenes* T-DNA [20]. RolD expressed by itself in transgenic plants induced early flowering and enhanced defense response [21,22]. *In vitro* assays demonstrated that RolD had ornithine cyclodeaminase activity [8]. The phenotypes of *RolD* overexpression are consistent with other observations suggesting a role of proline in flowering [5,6,13,23].

Although effects of expressing *A. rhizogenes OCD* in plants have been demonstrated, there has been no report of a protein having OCD activity in higher organisms. However, several plant genomes contain genes annotated as OCDs including *Arabidopsis* (AT5G52810), rice (Os10g38930), maize (GRMZM2G125266), soybean (Glyma20g30280) and *Thellungiella halophila* (Thhalv10014103m.g). We are not aware of any experimental data on the roles of these putative plant OCDs and whether they indeed have OCD activity. We characterized the *Arabidopsis* OCD-like gene, which we refer to as *AtOCD* for convenience and clarity as it has no other established name, with emphasis on whether or not it influences low water potential induced proline accumulation and may have a role in acclimation to low water potential. *AtOCD* transcript was up-regulated by low water potential and AtOCD was predominantly localized in the chloroplast which is thought to be a probable location for proline synthesis during stress. Physiological analysis of *atocd* and *atocd/p5cs1-4* mutants as well as *AtOCD* RNA lines demonstrated that lack of *AtOCD* expression significantly increased in proline accumulation at low ψ_w_ in a manner that was independent of *P5CS1*. However, we did not detect any OCD activity in biochemical assays with bacterially expressed AtOCD or AtOCD expressed in transgenic plants. Specific sequence differences between AtOCD and bacterial OCDs which have been structurally characterized also suggest that AtOCD may not catalyze the conversion of ornithine to proline. The data suggest that *AtOCD* is different than the characterized bacterial OCDs and has a unique function.

## Results

### AtOCD expression is up-regulated at low water potential

**Figure 1:**
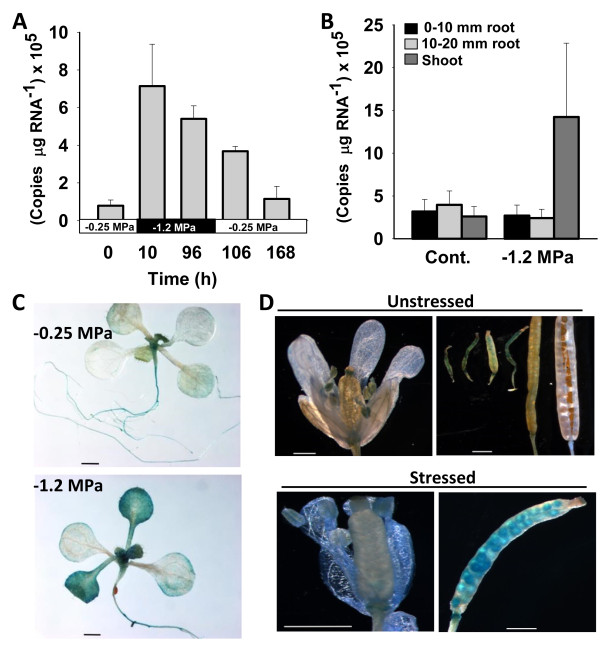
**Expression of *AtOCD *in response to low water potential stress. A**. QPCR analysis of transcript accumulation of *AtOCD* transcript levels after 10 or 96 h of low water potential stress. After 96 h of stress, seedlings were transferred back to the high water potential control (−0.25 MPa) for 10 or 96 h. Data are means ± SE (N = 5-6). **B**. Root versus shoot expression of *AtOCD* in control and −1.2 MPa treatments. Data are means ± SE (N = 5-6). **C**. Histochemical GUS staining of T_3_ Homozygous *AtOCDpromoter:GUS* transgenic plants expressing GUS under control of the 1.5 kb promoter region of *AtOCD*. Seven day old seedlings were transferred to −1.2 MPa stress treatment up to 96 h. Three independent transgenic lines were analyzed and representative images are shown. Scale bar in each panel equals 1 mm. **D**. GUS staining of floral organs of *AtOCDpro::GUS* plants under normal growing conditions or after water withholding. Scale bar in each panel equals 1 mm.

As a first step to determine the physiological function of the Arabidopsis OCD-like gene (*At5g52810,* referred to here as *AtOCD*), we examined its transcript levels under low water potential stress by transferring 7 day old seedlings to low water potential (−1.2 MPa) PEG-infused agar plates for 96 h. After 96 h of stress treatment, some seedlings were transferred back to control plates (−0.25 MPa) for stress release. *AtOCD* was up-regulated seven-fold within 10 h of low ψ_w_ treatment and expression remained high at 96 h (Figure 1A). During stress release, *AtOCD* level was down-regulated and returned to the basal level of expression. The observations are consistent with publically available microarray data which has also shown induction of *AtOCD* in response to various stress stimuli.

We have recently shown that other proline metabolism enzymes have tissue specific expression patterns at low ψ_w_[9]. We found that *AtOCD* was induced by low water potential specifically in shoot tissue while the root maintained a lower basal level of expression in both control and stress treated seedlings (Figure 1B). The expression pattern of *AtOCD* was similar to that of the proline biosynthetic gene *P5CS1* which had a greater low water potential induction in shoot than in root tissue [9]. To better define the tissue specific expression pattern of AtOCD, we generated transgenic plants with a GUS reporter driven by the 1.5 kB promoter region of *AtOCD. AtOCD* promoter activity was low in unstressed plants with some expression in root and localized expression in the shoot meristem and developing leaf tissue (Figure 1C). After 96 h at −1.2 MPa, *AtOCD promoter:GUS* expression was dramatically increased in shoot tissue with young leaves showing the greatest increase in expression. Thus, the GUS staining patterns agreed with the quantitative PCR results and suggested that AtOCD expression was greatest in shoot tissue at low water potential. In adult plants, water withholding induced *AtOCD promoter:GUS* expression in developing siliques and flowers (Figure 1D). No expression was seen in the mature seeds.

### AtOCD expression is induced by proline but not by its putative substrate ornithine

**Figure 2:**
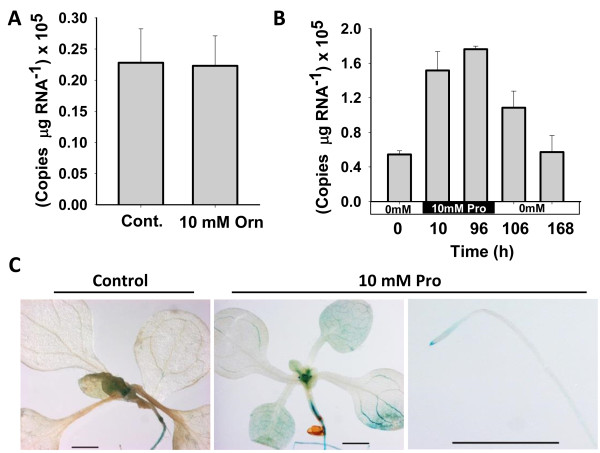
***AtOCD *expression in response to 10 mM Orn and Pro. A**. QPCR analysis of *AtOCD* expression after 96 h of exogenous ornithine treatment. Data are means ± SE (N = 5-6). **B**. *AtOCD* transcript accumulation after 10 and 96 h of 10 mM Pro treatment followed by removal of proline for 10 or 96 h. Data are means ± SE (N = 3–4). **C**. Histochemical GUS staining of T_3_ homozygous *AtOCDpromoter:GUS* transgenic plants treated with 10 mM Pro for 96 h. Three independent T3 homozygous lines were analyzed and representative images are shown. Scale bar in each panel equals 1 mm.

Since ornithine is a main substrate of bacterial OCD [8,17], we determined the effect of Orn on *AtOCD* expression by treating *Arabidopsis* seedlings with 10 mM Orn. No change in *AtOCD* transcript was observed between control and Orn treated seedlings (Figure 2A). In contrast, *AtOCD* level was up-regulated more than 3-fold after treatment with 10 mM proline and returned to the basal level after seedlings were removed from the proline-containing media (Figure 2B). Consistent with the quantitative PCR results, *AtOCD promoter: GUS* transgenic lines had increased GUS staining in response to Pro including expression in both shoot and root (Figure 2C). While there was an induction of *AtOCD* expression by proline, both quantitative RT-PCR and GUS-staining indicated that the induction was less than that caused by low water potential. We also tested 5 mM glutamate and 10 mM alanine and found no induction of *AtOCD* expression. Thus, *AtOCD* expression was specifically induced by proline. This is perhaps consistent with the fact that there is a proline response element (ACTCAT; [24]) at −574 bp of the *AtOCD* promoter [25].

### AtOCD is predominantly localized in plastids

**Figure 3:**
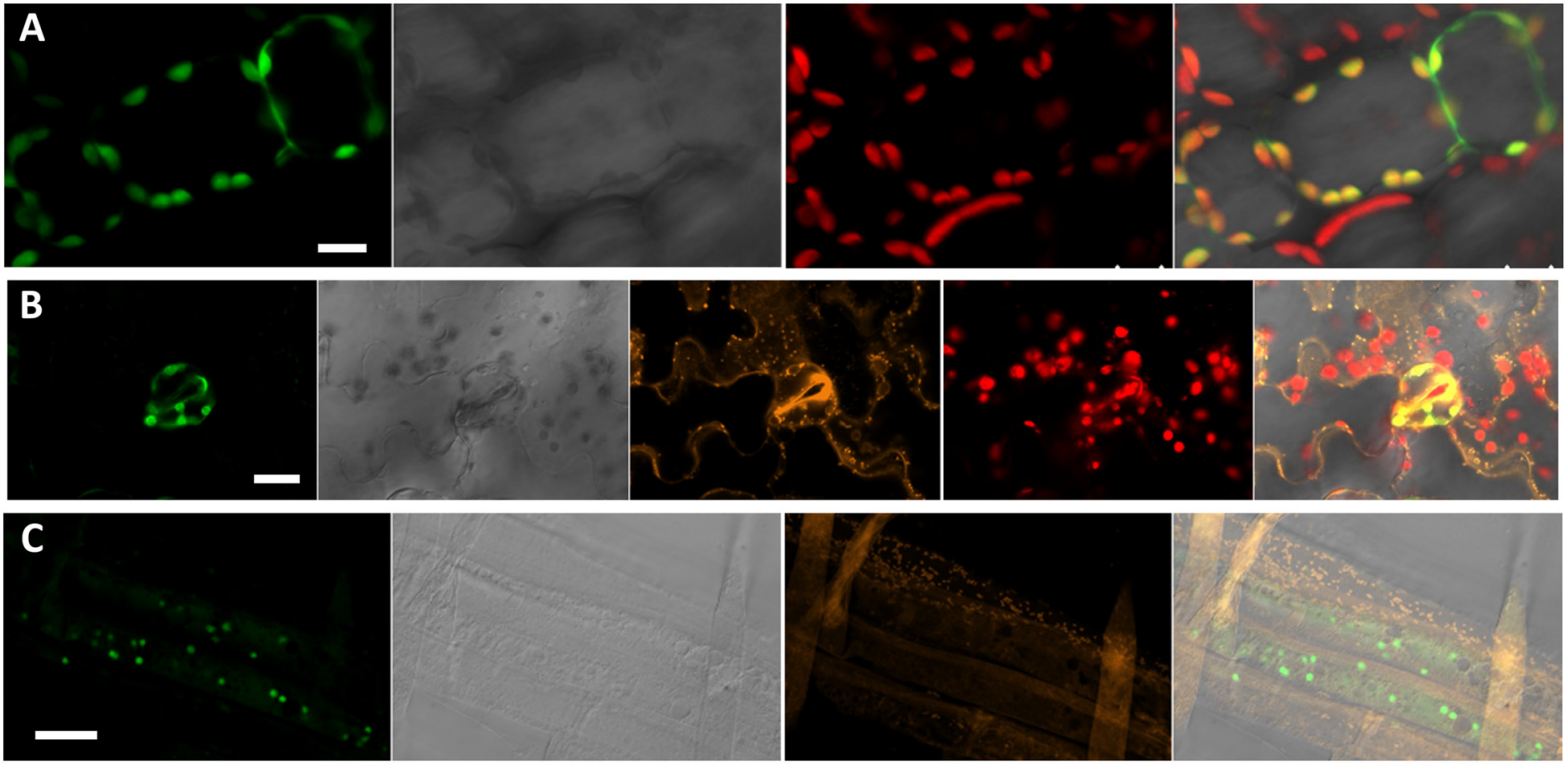
**Subcellular localization of AtOCD.** Four to ten day old *Arabidopsis* seedlings expressing OCD-EYFP fusion construct under the control of CaMV 35S promoter were examined by confocal microscopy. **A**. Hypocotyl cells. The images are (from left to right) EYFP fluorescence, bright field image, chlorophyll fluorescence and overlay of all three images. Scale bar indicates 10 μm. **B**. Leaf epidermis showing AtOCD expression in a guard cell. The images are (from left to right) EYFP fluorescence, bright field image, mitotracker staining, chlorophyll fluorescence and overlay of all images. Scale bar indicates 20 μm. **C**. Cells from just behind the cell elongation zone in the root (note root hairs that can also be seen in the images. The images are (from left to right) EYFP fluorescence, bright field image, mitotracker staining, and overlay. Scale bar indicates 20 μm.

To determine the subcellular localization of AtOCD, we generated transgenic plants expressing a C-terminal fusion of AtOCD to EYFP. Most, but perhaps not all, of AtOCD:EYFP fluorescence co-localized with chlorophyll auto-fluorescence in hypocotyl cells (Figure 3A), and stomata (Figure 3B). In roots (Figure 3C), AtOCD localized to structures that are presumably proplastids as they were larger and fewer in number than mitochondria labeled by Mitotracker staining. Together these data indicated a predominantly chloroplast localization of AtOCD and is consistent with its predicted localization. As proline synthesis during stress may occur in the chloroplast [1,2], the AtOCD localization was not inconsistent with a connection of AtOCD to proline synthesis.

### ***atocd*** mutants and RNAi lines have increased proline content at low ψ_w_ but are not affected in growth nor in flowering time

**Figure 4:**
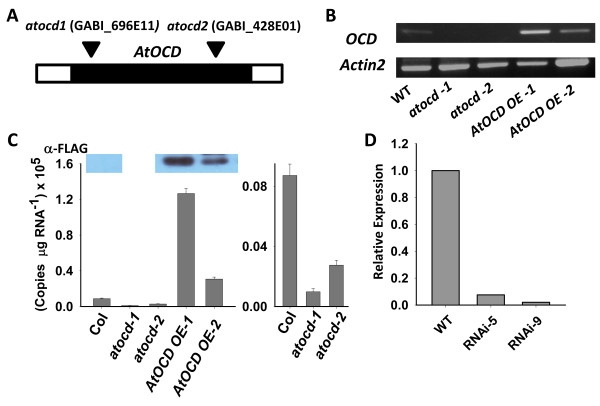
**Characterization of *atocd *mutants and over-expression lines. A**. Schematic representation of *AtOCD* to show the position of T-DNA insertions. Black bar indicates coding region while unfilled bars represent 5′ and 3′ untranslated regions. **B**. Semi-quantitative RT-PCR to show the *AtOCD* transcript accumulation in WT, mutants and over-expression lines. *Actin2* gene is used as a positive control. **C**. Quantitative real time PCR of *AtOCD* in mutant and overexpression lines. Error Bars indicate S.E. of triplicate samples. Panels at the top of the figure show western blotting of wild type and AtOCD overexpression plants to detect the C-terminal FLAG tag. All lanes were on the same gel and are separated for clarity of presentation. The band shown migrated at the expected molecular weight for the AtOCD-FLAG fusion protein (37.7 kD). Right panel of the figure shows the wild type and *atocd* mutant results on an expanded scale to show clearly the greatly reduced mRNA level in the *atocd* muants. **D**. Relative expression of *AtOCD* in two independent *AtOCD* RNAi lines. RNA was isolated from unstressed seedlings.

To investigate the physiological function of *AtOCD*, we isolated homozygous lines for two *AtOCD* T-DNA insertion mutants, *atocd-1* (GABI_696E11) and *atocd-2* (GABI_428E01) (Figure 4A) and demonstrated that both lacked *AtOCD* transcript (Figure 4B and C). RNAi knockdown lines of AtOCD were also generated and shown to have decreased *AtOCD* expression (Figure 4D). Plants expressing FLAG-tagged *AtOCD* under control of the 35S promoter were also generated and confirmed to express AtOCD-FLAG protein (Figure 4C).

**Figure 5:**
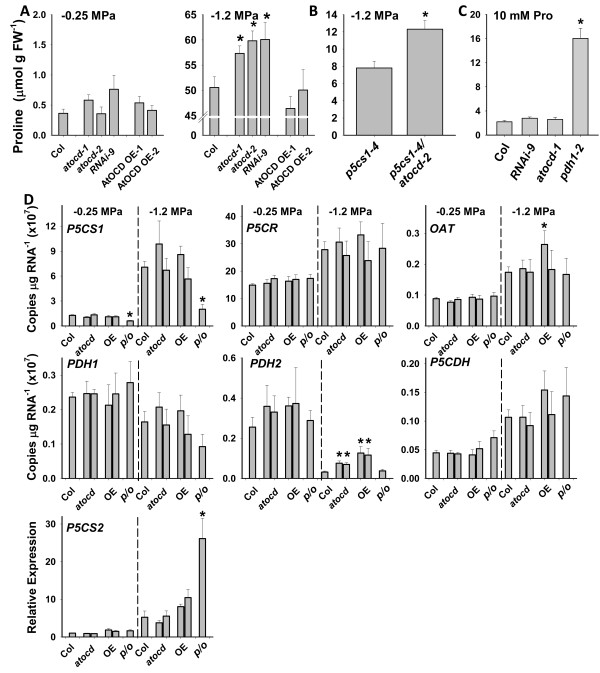
**Proline content in *AtOCD *genotypes in response to low water potential or exogenous proline and proline metabolism gene expression. A**. Seven-day-old seedlings of *AtOCD* mutants, RNAi and over-expression lines were transferred either to unstressed (−0.25 MPa), or to PEG-infused agar plates to impose low water potential (−1.2 MPa) for 96 h. Data are means ± S.E. (n = 6). One representative data set is shown out of four independent experiments performed. Asterisks indicate significantly different proline contents compared to the Col wild type (p ≤ 0.05). **B**. Proline content of *p5cs1-4* and ocd-2/*p5cs1-4* after 96 h at −1.2 MPa. Data are means ± S.E. (n = 6) combined from two independent experiments. Asterisks indicate significant difference between genotypes (p ≤ 0.05). **C**. Seedling proline content 96 h after transfer of seven-day-old seedlings to media containing 10 mM proline. Data are means ± SE (N = 3-4). **D**. Proline metabolism gene expression for seven-day-old seedlings transferred to either −0.25 MPa or −1.2 MPa media for 96 h. Data are means ± SE (N = 6) combined from two experiments. Asterisks indicate significant difference of mutant or overexpression line compared to Col wild type at the same water potential. In the axis labels, *atocd* indicates the *atocd* T-DNA mutants (*atocd-1* left bar and *atocd-2* right bar), *OE* indicates *AtOCD* overexpression lines (*AtOCD OE-1* left bar, *AtOCD OE-2* right bar) and *p/o* indicates the *p5cs1-4/atocd-2* double mutant.

To determine the role of *AtOCD* in proline accumulation, seedlings of each genotype were transferred to unstressed (−0.25 MPa) or stress (−1.2 MPa) treatments for 96 h. This length of treatment was used as previous observations in our laboratory found that proline accumulates steadily after transfer to low water potential and reaches a maximal, near steady state level at 96 h [14]. No significant difference in proline content was shown by any of the *AtOCD* genotypes under control conditions; however, at −1.2 MPa, a significant increase in proline accumulation was observed in both *atocd* mutants and RNAi lines compared to wild type (Figure 5A). As a percentage of the total proline accumulation, the effect was moderate (12-17%); however, as 96 h low water potential treatment elicits very high levels of proline accumulation, the absolute amount of proline increase (5–7 μmol g FW^-1^) was substantial. In contrast, overexpression of *AtOCD* did not affect proline accumulation.

It is well established that most of the low water potential induced proline accumulation is dependent on proline synthesis by *P5CS1*[9,14]. To determine if increased proline caused by lack of *AtOCD* expression was also dependent on *P5CS1*, we generated a *p5cs1-4/atocd-2* double mutant. Consistent with our previous results, proline content of *p5cs1-4* was only 15% that of the wild type at low water potential [9]. Proline content of the *p5cs1-4/ocd-2* double mutant was increased by nearly 5 μmol g FW^-1^ (Figure 5B), similar to the amount of increase seen in the *atocd* single mutant relative to wild type. In relative terms, this was a nearly 40% increase in proline content of the double mutant compared to the *p5cs1-4* single mutant. These results suggested that AtOCD did not contribute to stress-induced proline accumulation as may be expected if it catalyzed the conversion of ornithine to proline. Instead, it had a negative effect on proline accumulation that was independent of P5CS1. We also assayed proline content of seedlings after application of exogenous proline to determine whether AtOCD may contribute to proline catabolism (Figure 5C). No difference was observed between wild type and atocd mutant or RNAi seedlings while *pdh1-2* mutants inhibited in proline catabolism accumulated much higher levels of proline, consistent with previous experiments [14]. These data suggested that AtOCD does not substantially contribute to proline catabolism.

One possibility is that suppression of AtOCD affected proline levels by altering expression of proline metabolism genes. We assayed the expression of proline synthesis genes *P5CS1* (*At2g39800*)*, P5CS2* (*At3g55610*) and *P5CR* (*At5g14800*) as well as the proline catabolism genes *PDH1* (*At3g30775*)*, PDH2* (*At5g38710*) and *P5CDH* (*At5g62530*). We also assayed ornithine amino transferase (*OAT, At5g46180*), which can synthesize proline *in vitro* but may not contribute to proline synthesis *in planta*[26]. The effect of low water potential on expression of these genes in wild type was consistent with previous observations in our laboratory [14] and many other reports in the literature. In comparing the *AtOCD* mutants and transgenic lines to wild type, we found no substantial difference in expression of proline synthesis genes that could explain the higher proline of the *atocd* mutants (Figure 5D). The only difference in proline synthesis gene expression that we observed was increased *P5CS2* in *p5cs1-4/atocd-2*. As *P5CS2* expression was unaffected in any of the *AtOCD* mutants or transgenic lines, this likely was a compensation for the much reduced proline level caused by the lack of *P5CS1* expression. Note that *p5cs1-4* still accumulates a reduced level of truncated P5CS1 transcript but has been shown to be a null mutant that does not produce P5CS1 protein [11,27].

For proline catabolism genes the only difference observed was increased expression of *PDH2* in *atocd* mutants and an even greater (nearly 4-fold increase) in the *AtOCD* overexpression lines at low water potential (Figure 5D). The significance of this is not clear as the *AtOCD* overexpression lines had the highest expression of *PDH2* but did not significantly differ from wild type in proline accumulation (Figure 5A). In the *atocd* lines, increased *PDH2* expression may be an indirect effect of the higher proline level. *OAT* was slightly more expressed in one of the overexpression lines at low water potential (Figure 5D) and this line also had the highest expression of *AtOCD* (Figure 4B). This suggests an effect of AtOCD overexpression on mitochondrial metabolism as both PDH2 and OAT are mitochondrial proteins [26,28]. Overall, the gene expression results suggested that AtOCD may affect proline metabolism indirectly or through post-transcriptional mechanisms as there was no change in gene expression that could simply explain the higher proline in *atocd* mutants.

**Figure 6:**
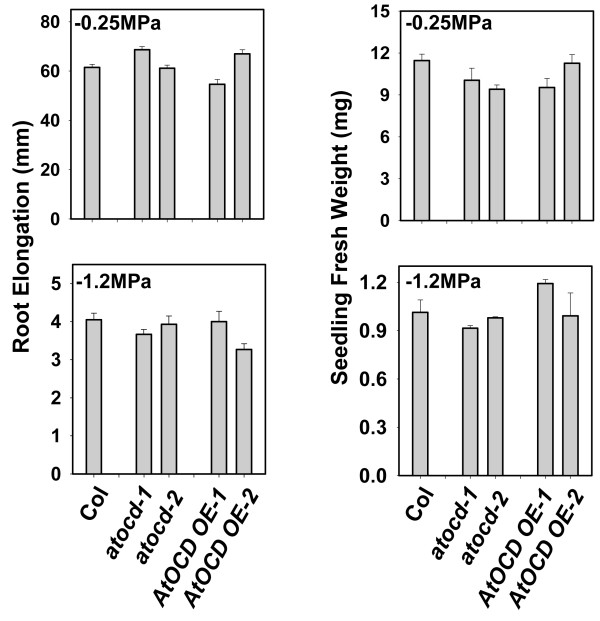
**Growth of *AtOCD *mutants and over expression lines at low ψ**_**w**_**.** Five-day-old seedlings were transferred to −1.2 MPa PEG-infused agar plates or fresh control (−0.25 MPa) plates and root growth and fresh weight measured ten days of transfer. Data are means ± SE (N = 2-3) for fresh weight and (N = 8–15) for root elongation. No significant differences were found between mutants or transgenics versus wild type.

**Figure 7:**
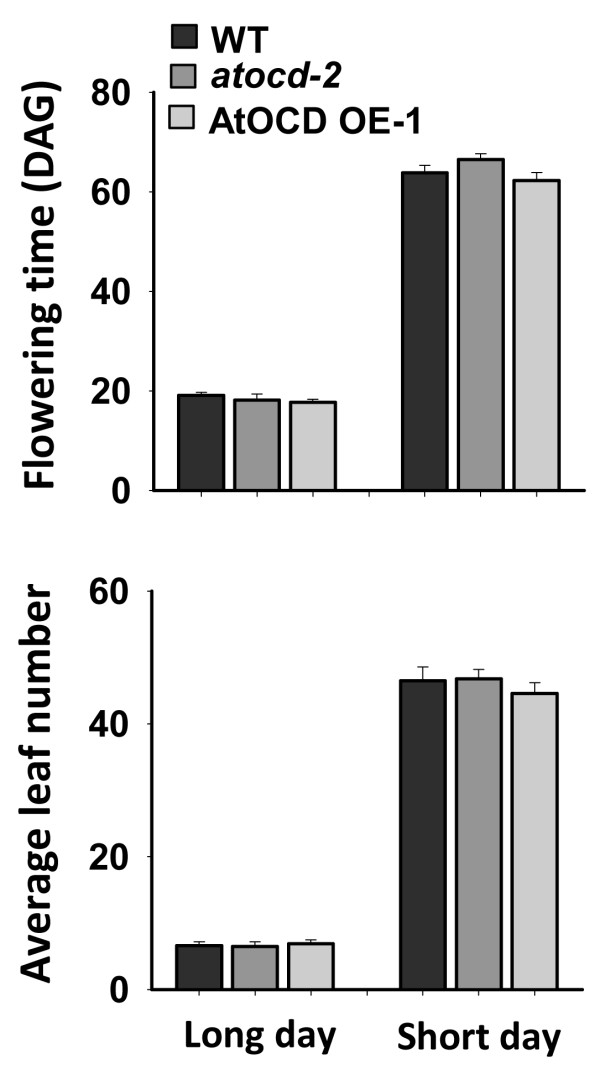
**Analysis of flowering time in WT, *atocd *and *AtOCD *overexpression lines under short or long day growth conditions.** Flowering time was scored based on the emergence of the first floral bud. Short day plants were kept under an 8 h light/16 h dark cycle while long day plants were kept under a 16 h light/8 h dark cycle. No difference in the flowering time was observed. Data are means ± SE (N = 5-10).

Both *p5cs1* and *pdh1*, which are inhibited in proline synthesis or proline catabolism respectively, have decreased root elongation and seedling fresh weight when exposed to a controlled severity of low water potential stress using PEG-infused agar plates [9]. These observations demonstrated an importance of both proline synthesis and catabolism in low water potential tolerance. Similar phenotypic assays of *AtOCD* mutants or overexpression lines found no difference in root elongation or seedling fresh weight under either control or low water potential conditions (Figure 6). The flowering time of the *AtOCD* mutants and transgenics was also of interest since expression of the *A. rhizogenes rolD* OCD induced early flowering in transgenic plants [8,22]. However, no significant difference in the flowering time was shown by any of our At*OCD* genotypes in either short or long day growth conditions (Figure 7). Flowering times were counted based on appearance of the first floral bud and were similar to previous report [6].

### In vitro activity assays and sequence analysis of AtOCD

**Figure 8:**
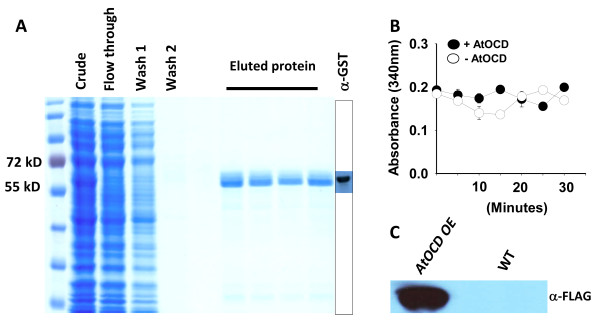
**Expression and purification of recombinant AtOCD. A**. Commasie blue stained SDS-PAGE gel showing different purification steps of GST fused AtOCD and western blot using anti GST antibody to confirm the identity of the GST-AtOCD band. **B**. Time course analysis with purified AtOCD protein showed no change in the absorbance reading after 30 min of incubation with the putative substrate ornithine and NAD^+^. Data are representative results of one out of 3–4 independent experiments using independent purifications of recombinant AtOCD. Data are means ± SE (N = 2-3) of replicate assay tubes. Similar results were obtained either from bacteria or plant purified AtOCD protein and using different substrate and cofactor combinations described in the text. **C**. AtOCD fused with FLAG immunoprecipitated from T_3_ transgenic plants and detected by western blot. A mock purification from wild type plants was used as a control. The band migrated at the expected size of AtOCD:Flag (37.7 kD).

Activity assays using recombinant AtOCD protein produced in *E. coli* (Figure 8A) were performed as previously described [8,18] using ornithine as a substrate and either NAD or NADP as the cofactor. No activity was observed in repeated assays and using several batches of independently purified recombinant protein (an example of activity assay data is shown in Figure 8B). Since bacterial expressed protein may lack post-translational modifications necessary for activity, we also immunoprecipitated FLAG-tagged AtOCD from transgenic Arabidopsis plants. Successful pull down of tagged protein was confirmed by western blot (Figure 8C); however, we were again unable to detect OCD activity in assays using the immunoprecipitated protein. Additional assays with the ornithine and proline related amino acids γ-amino butyric acid (GABA), glutamate and glutamine also did not detect any activity. Also, in at least in few bacteria, OCD can catalyze the reverse reaction to produce ornithine from proline [29]. We also tested proline as a substrate for AtOCD but could not detect any activity. These assays suggested that AtOCD does not have ornithine cyclodeaminase activity; although, we of course cannot rule out the possibility that some factor or modification of the AtOCD protein needed for activity was missing in our assays.

**Figure 9:**
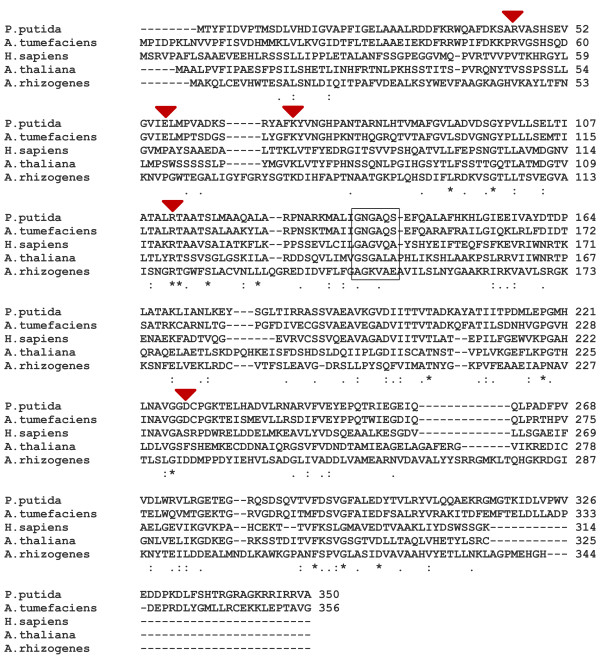
**Amino acid sequence alignment of bacterial OCDs, human mu-crystallin, and AtOCD.** Conserved sequences essential for L-Orn binding of bacterial OCDs are indicated with red triangles. *Peudomonas putida* Asp 228, which directly interacts with the leaving α- amino group, is also indicated with a red triangle. Box indicates the conserved sequence of the Rossman NAD- binding domain (GxGxxG/A). Protein accessions numbers are: *Peudomonas putida* (NP_745670), *Agrobacterium tumefaciens* (AAF77139.1), Homo sapiens (AAH18061.1) and Arabidopsis thaliana (AED96264). A rhizogenes OCD sequence is from Trovato et al. [13].

To further understand these results, we examined the AtOCD sequence in comparison to proteins confirmed to have OCD activity and human mu-crystallin which is structurally related to OCD. The crystal structure of *Pseudomonas putida* OCD has been resolved and used to determine the key amino acid residues required for binding to L-ornithine [30]. This structural analysis revealed that Arg45, Lys69, Arg 112 of *P. putida* OCD were critical for interaction with the L-ornithine carboxyl group. Also in *P. putida* OCD, Glu56 his critical in substrate binding and Asp228 directly interacts with the α-amino leaving group during the deamination of ornithine. Several of these critical residues (Arg45, Glu56 and Asp228) are not conserved in AtOCD nor in human mu-crystallin which has a different, although not well understood, function than bacterial OCDs (Figure 9). Interestingly, the sequence of *A. rhizogenes* OCD reported by Travato et al. [8] is an outlier as it differs from both the *P. putida* and *A. tumefacians* OCDs as well as AtOCD at these critical amino acid residues (Arg45 and Glu56). As no structural information is available for *A. rhizogenes* OCD, the implications of its differing sequence are unclear. Nonetheless, these sequence differences along with our inability to detect activity of AtOCD argue against it having the same enzymatic function as bacterial OCDs.

### Metabolic profiling identifies metabolite changes induced by low water potential but relatively few differences in AtOCD mutants or overexpression lines

To further characterize the function of AtOCD, seedling samples of wild type and *AtOCD* mutant or overexpression lines were subjected to GC-MS untargeted metabolite analysis. Our hypothesis was that candidate substrates or products of AtOCD would have their relative abundance affected oppositely by *AtOCD* overexpression versus *atocd* knockout (for example, a metabolite more abundant in *AtOCD* overexpression plants but less abundant in *atocd* mutant). As *AtOCD* expression was induced by low water potential, we also hypothesized that AtOCD substrates or products may accumulate at low water potential.

In the metabolite data set, 471 metabolites consisting of both known compounds and those identified only as mass spectral tags [31] were quantified. A number of metabolites were found to accumulate in wild type at low water potential including several unidentified compounds (See Additional file 1: Table S1). A forty eight-fold accumulation of proline was observed (one clearly outlying control sample was removed in this case) which matches well with the proline content accumulated in wild type after 96 h of −1.2 MPa (Figure 5B). In addition, an increase in isoleucine and leucine was observed, consistent with observations in our laboratory using targeted metabolite analysis by GC-MS [32]. As for the *AtOCD* overexpression and knockout data, there were relatively few differences in metabolite content and no metabolite that clearly met the above criteria for an AtOCD substrate or product. Particularly, no compound had a clearly opposite change in abundance in the *atocd* mutant versus *AtOCD* overexpression line. One unidentified metabolite (number 616767) which accumulated approximately 6-fold in wild type at low water potential was also increased approximately 5-fold in *AtOCD* overexpression plants under control or stress conditions but was not affected in *atocd* (Additional file 1: Table S1). A few other unknown compounds were more or less abundant in both *AtOCD* overexpression plants and *atocd* mutant. Of known compounds, ornithine was found to be higher in overexpression lines at low water potential; however, we were unable to detect any activity with ornithine or with proline as a substrates (see above). Interestingly, ascorbate was the known compound most affected in the AtOCD lines: it was lower in both *AtOCD* overexpression and *atocd* mutant samples. This difference may represent an indirect effect of *AtOCD* expression on plant redox status.

## Discussion

We studied an uncharacterized *Arabidopsis* protein having sequence similarity with bacterial OCDs which produce proline in a single step by deamination of ornithine [18]. The existence of such direct ornithine to proline conversion in plants could potentially by pass the main pathway of proline synthesis in which glutamate is converted to proline via the intermediate P5C. As P5C has been proposed to affect ROS production or potentially act as a signaling molecular in its own right, a pathway that bypassed P5C would be of great interest. Our data do not allow us to conclusively rule out such a pathway in plants, but the phenotypic assays, *in vitro* enzyme assays and sequence differences between the Arabidopsis OCD-like protein (AtOCD, At5g52810) and structurally characterized bacterial OCDs all suggest that AtOCD does not synthesize proline. Our results are consistent with the findings of Trovato et al. (2001), who concluded that OCD activity was not present in plant extracts [8].

Despite its apparent lack of OCD activity, *AtOCD* does have some effect on proline metabolism as both *atocd* mutants and RNAi lines as well as a *p5cs1-4/atocd-2* double mutant had increased proline. The observation that proline, but not other amino acids, could induce *AtOCD* expression also suggests a connection of *AtOCD* to proline metabolism. One possibility is that a lack of AtOCD activity changes cellular redox status and that this altered redox status causes a change in proline metabolism. It has been observed that proline metabolism is required to buffer NADP^+^/NADPH ratio [9] and that lack of *P5CS1* expression changes reactive oxygen metabolism [11]. Thus, if *AtOCD* expression has an effect on redox status, this could in turn affect proline. This idea is supported by the observation that manipulation of *AtOCD* expression decreased the level of ascorbate (Additional file 1: Table S1). Of the proline metabolism genes we assayed, only *PDH2* and *OAT* showed any significant change in expression in *AtOCD* mutant or transgenic lines. This observation that plastid-localized *AtOCD* affected the expression of mitochondrial localized *PDH2* and *OAT* also suggests an indirect mechanism for the altered proline accumulation in *atocd* mutants. It also indicates, consistent with ongoing work in our laboratory, that there are many ways to alter proline accumulation other than transcriptional regulation of proline metabolism genes. Altered protein levels, enzyme activity, or substrate availability are all possibilities. We also cannot rule out the possibility that AtOCD activity somehow competes with proline metabolism. However, in that case it is unclear why the effect of *atocd* is the same in wild type and the *p5cs1-4* mutant background where total proline synthesis is much less.

OCD-like proteins have been identified in several organisms based on bioinformatic prediction however; like AtOCD, none of them showed OCD activity due to lack of conserved substrate binding sites [19,33-36]. The function of these OCD-like proteins is largely unknown although they have been associated with a number of phenotypes. For instance, an OCD-like protein in *Rhizobium melliloti* was associated with enhanced nodulation efficiency [36]. In *Halichondria japonica,* another OCD orthologue functions as a tauropine dehydrogenase involved in opine metabolism [34] and in *Archaeoglobus fulgidus*, an OCD-like protein functions as an alanine dehydrogenase [33]. These examples suggest that OCD-like proteins are functionally divergent and have evolved to play specific roles in each of these organisms.

Recent observations have shown a role of proline metabolism in plant-pathogen interaction [1,10]. Also, proline is involved in the activation of quorum sensing and antagonizes the effect of GABA in plant defense response against *A. tumefaciens* infection [37]. Interestingly, *AtOCD* is differentially expressed between *Arabidopsis* accessions that demonstrate varying susceptibility levels to pathogen infection [38]. This suggests a possible role for AtOCD in plant defense responses. However, preliminary tests of our *AtOCD* overexpression lines did not reveal any difference in susceptibility when the plants were challenged with *Pseuodomonas syringae* p.v. tomato (DC3000). Whether or not AtOCD may participate in some aspect of secondary metabolism related to biotic stress remains a possibility.

## Conclusions

A combination of physiological, biochemical and genetic analyses found that AtOCD does affect proline accumulation at low water potential via a mechanism that is independent of P5CS1. The effect of AtOCD on proline accumulation may be indirect, possibly reflecting a change in redox status. The data suggest that AtOCD may have functions and substrate preference different than previously characterized bacterial OCDs.

## Methods

### Plant material, growth conditions and stress treatments

T-DNA insertion lines of Arabidopsis OCD-like gene *At5g52810* (GABI_428E01 and GABI_696E11) were obtained from the GABI-KAT collection and genotyped by PCR. Mutants of *P5CS1* (*p5cs1-4*) and *PDH1* (*pdh1-2*) have been previously characterized by our laboratory and others [9,11,14]. Screening for *atocd-2/p5cs1-4* double mutants was conducted using primers given in Additional file 1: Table S2 and those reported previously for *p5cs1-4*[14]. For plate based experiments, seeds were surface sterilized and grown in half strength MS medium (with 2 mM MES buffer pH 5.7, no sugar added). After two days of stratification, plates were transferred to a growth chamber (25°C, continuous light at 80–100 μmol photons m^-2^ s^-1^) and incubated vertically. Seven-day-old seedlings were subjected to low water potential stress by transferring them to agar plates infused with PEG-8000 [39]. For amino acid treatment, proline, ornithine, or other amino acids were added to the medium after sterilization at the concentrations indicated in the text or figures. Water stress treatment of soil grown plants was given by withholding water for 10 days.

### Generation of transgenic plants

To generate *AtOCD* RNAi lines, a 400 bp portion of *AtOCD* was amplified from Col-0 genomic DNA and cloned into entry vector pDONR207 by BP reaction (Invitrogen). The AtOCD sequence used for RNAi was chosen based on the Agrikola database of gene specific tags [40]. The insert was transferred into pHELLSGATE12 [41] through LR reaction and the construct was sequenced to confirm orientation of the inserts. Transgenic plants were generated using *Agrobacterium tumefacians* strain GV3101 and floral dip transformation [42]. Transgenic lines were selected on media containing 50 μg ml^-1^ kanamycin and two or three independent T_3_ homozygous lines were used for phenotypic assays.

To generate *promoter:GUS* lines, a 1.5 kB upstream promoter region of *AtOCD* was amplified from Col-0 genomic DNA. A second nested PCR was done to add the remaining portion of the Gateway sequences and Gateway recombination reactions were used to clone the fragment into pDONR207 and subsequently into pGWB433 [43]. Three independent homozygous T_3_ lines were used for GUS staining with representative images shown. For overexpression, the open reading frame of *AtOCD* was amplified without stop codon and cloned into pDONR207. The inserts were transferred by LR into destination vectors pGWB411 or pGWB441 for expression of AtOCD with C-terminal FLAG or EYFP tag, respectively [43]. All primers used for cloning can be found in Additional file 1: Table S2.

### RT-PCR and quantitative real time PCR

Total RNA was isolated using RNAeasy plant mini kit (Qiagen) with DNAse treatment. RNA was quantified by nanodrop spectrophotometer and 1 μg total RNA reverse transcribed using SuperScript III (Invitrogen). *Actin2* was used as a reference gene for semi-quantitative RT-PCR. Quantitative real time PCR was performed using a FAM/BHQ labeled TaqMan probe and primer set (Additional file 1: Table S2). Absolute quantitation of copy numbers of *AtOCD* and proline metabolism genes was based on a standard curves constructed using plasmid DNA. For proline metabolism genes, primer and probe sets, RT-PCR and quantitation were performed as previously described [14]. *P5CS2* was quantified using the ∆∆C_t_ method using *Actin8* as a reference gene and primers given in Additional file 1: Table S2. Three to six independent samples were used for each gene expression measurement. Tissue specific gene expression analysis was carried out with shoot tissue and two different 10 mm root sections collected as previously described [9].

### Microscopy

For confocal microscopy analysis, 4–10 day old seedlings mounted in water were examined with a 63 × /1.2 W Korr UV–VIS-IR M27 water immersion lens on a LSM510 META laser-scanning confocal microscope (Carl Zeiss Inc., Thornwood, NY, USA). Fluorescence images were obtained at 1024 × 1024 pixel resolution by using the 514-nm excitation line of an Argon/2 ion laser (458, 477, 488 and 514 nm) with appropriate emission filters for YFP and chlorophyll. For *in vivo* mitochondrial staining seedlings were mounted in water and supplemented with 1 mM of MitoTracker® Orange CM-H_2_TMRos (M-7511, Molecular Probes, Inc., Eugene, OR, USA). Images were then acquired using two channels with separate excitation by 514 nm (YFP) and 543 nm (MitoTracker Orange) laser lines, and fluorescence emissions were gathered. Images were exported as TIFF files and further processed with LSM 5 META Image Examiner.

### Protein expression, purification and enzyme assay

The *AtOCD* open reading frame was transferred into pDEST15 (Invitrogen) by LR reaction to express GST-tagged AtOCD. The construct was transformed into Rosetta (Merck) *E. coli* cells. The bacterial cells was lysed in 1X GST binding buffer (43 mM Na_2_HPO_4_, 14.7 mM KH_2_PO_4_, 1.37 mM NaCl and 27 mM KCl, pH 7.3), 100 μg/ml lysozyme, 1 mM PMSF, 0.1% Triton and 1X protease inhibitor (Roche) and incubated for 30 min at room temperature. The cell lysate was sonicated (10 s pulse cycle for 1 min) and centrifuged at 12,000 g for 15 min at 4°C. The recombinant protein was expressed in soluble form with the expected size of ~ 62 kD and was purified with GST binding resin (Novagen) and eluted in 50 mM Tris-Cl (pH 8) and 100 mM Glutathione. Excess glutathione was removed by overnight dialysis against 10 mM Hepes (pH 8.2), 10 μM NAD^+^, 50 mM NaCl and 1X protease inhibitor (Roche) by changing the buffer several times at 4°C. Purified protein was detected on SDS gel and confirmed by western blot with GST antibody.

Alternatively AtOCD:Flag was immuno-precipitated from transgenic plants. Briefly, transgenic and untransformed (control) plant tissue (20 g) were homogenized in lysis buffer (10 mM Hepes (pH 7.5), 10% Glycerol, 10 mM KCl, 5 mM MgCl_2_, 100 mM β-mercaptoethanol, 1 mM PMSF and 1X protease inhibitor (Roche) and the crude homogenate centrifuged. The supernatant was incubated with anti-Flag resin (Sigma) for 3–4 h. The anti-FLAG resin was collected by low speed centrifugation and washed 3 times with lysis buffer. The resin, suspended in a small volume of lysis buffer, was transferred into Pierce spin cups (Thermo Scientific), incubated with 3X FLAG Peptide (Sigma; 25 μg in 100 μl lysis buffer) for 20 min and protein eluted in lysis buffer or in 10 mM Hepes (pH 8.2), 10 μM NAD^+^, and 50 mM NaCl and 0.2 mM PMSF and protease inhibitor (Roche). All purification steps were carried out in a cold room. Protein samples were divided into single use aliquots and stored into −80 C.

Enzymatic assay of purified *AtOCD* was performed using similar conditions as reported [8,18] with some modifications. The reaction mixture consist 10 mM Hepes (pH 8.2), 5 mM Orn, 2 mM NAD^+^, 1 mM DTT and 100–200 ng AtOCD protein in 200 μl total volume. Other co-factors including NADP, NADPH and NADH and additional possible substrates such as glutamate, GABA and alanine were also used in different combinations but keeping all concentrations the same. The reverse OCD reaction was assayed using 10 mM Hepes (pH 8.2), 5 mM Pro, 1 mM NADH and 700 mM NH_4_Cl (final pH of the reaction was 7.6) [33]. The reaction was incubated at room temperature (25 C) and change in the absorbance was measured over 30 min in a plate reader.

### Proline measurement and metabolite profiling

Proline measurement was done by ninhydrin assay [44] with sample collection and extraction as reported in [9]. For metabolite profiling, samples (~80 mg) of unstressed seedlings or seedlings exposed to −1.2 MPa for 96 h on PEG-agar plates were collected and lyophilized. Sample extraction, GC-TOF-MS analysis and metabolite identification were performed at the UC Davis Genome Center Metabolomics Facility [45].

### Availability of supporting data

The data set supporting the results of this article is included within the article and its additional file.

## Competing interests

The authors declare that they have no competing interests.

## Author contributions

SSha performed research, designed experiments and wrote the paper; SShi performed research; PEV conceived the research, directed experiments and wrote the paper. All authors read and approved the final manuscript.

## Supplementary Material

Additional file 1: Table S1 Metabolite profiling of WT, atocd-2 and AtOCD OE: Three samples each of genotypes were collected from seedlings under control conditions or after 96 h at -1.2 MPa. Samples were sent to metabolomics core facility at the University of California-Davis for analysis by GC-MS. Metabolite abundances were calculated relative to internal standards and normalized to sample weight. Data shows the average normalized peak areas ± SE of metabolites in WT, atocd and AtOCD OE (Panel A and B) under control and stress treatments. The fold change difference in metabolite level between atocd versus wild type and AtOCD overexpression versus wild type was calculated (Panel C). The change in metabolite abundance in wild type under stress verus control treatment was also calculated. One control sample of the AtOCD over-expression line was lost during sample processing and thus these values are based on the average of two samples. Statistical analysis consisted of T-test comparison of the peak areas for each known compound among genotype/treatment combinations. Such analysis and was done for known metabolites. Bold values in Panel C indicate significant difference in metabolite abundance. **Table S2**: Primers used in this study. Click here for file
